# Factors influencing compliance of closed fishing season: lessons from small-scale coastal fisheries in the Central Region of Ghana

**DOI:** 10.1057/s41599-023-01513-4

**Published:** 2023-01-17

**Authors:** Victor Owusu, Kofi Adu-Boahen, Sender Kyeremeh, Innocent Demalie, Philip Eshun

**Affiliations:** Department of Geography Education, University of Education, Winneba, Ghana

**Keywords:** Geography, Development studies

## Abstract

This paper contributes to the literature on marine conservation and its implication for coastal governance and sustainability. The study investigates factors influencing fisherfolk compliance with the temporal fishing bans in Ghana. The purpose is to understand the factors influencing compliance behaviour to help design an improved conservation strategy to achieve management objectives. A mixed-method approach was employed, consisting of 200 household surveys and 17 in-depth interviews with stakeholders at the local, district, regional, and national levels. The results reveal that a combination of instrumental and normative factors influences fisherfolk’s compliance behaviour concerning the closed fishing season. Participation of fisherfolk and coastal communities in the fisheries management decision-making processes positively influenced compliance. The key drivers of non-compliance with the closed season emanate from a perceived lack of ecological effectiveness, lack of enforcement of sanctions, and lack of compensation for loss of income during the ban. The study suggests co-management that includes fisheries agencies and traditional authorities as a viable option for fisheries management and marine conservation initiatives. In addition, the paper recommends the introduction of conservation payment schemes alongside strict monitoring of the temporal ban on fishing.

## Introduction

Small-scale fisheries (SSF) are vital in providing food security in Africa. An estimate shows that approximately 200 million Africans rely on fish as their primary source of animal protein, with another 90 million depending on fisheries as part of a diversified livelihood strategy (Motta, 2015). In recent years, the SSF in Africa has suffered from rapidly declining marine fish landings resulting in decreased income and severe economic hardships (Hasselberg et al., 2020; World Bank, 2016). SSF refers to fishing and its related activities, such as processing and trading, operated by individuals and families in coastal communities and is characterized by low investment and technology (Owusu and Andriesse, 2022; Finegold et al., 2010).

In Ghana, the SSF classification is based on the type of gear the canoes operate; the most common are: purse seine, hook and line, drift gillnet, beach seine, and lobster set net. SSF target both pelagic and demersal species. The ones targeting high-value large aquatic and demersal species (e.g. yellowfin tuna, mackerel, sailfish, and shark) use hook and line and, drift gillnets. In contrast, others target mackerel, cassava fish, herrings, barracuda, and lobsters using pursing and lobster nets. Canoes with 25–40 horsepower outboard motors and electronic devices such as Global Positioning Systems (GPS) and echo sounders can travel further offshore to exploit high-value species. Fishers who travel further distances offshore use ice to preserve the fish caught whilst staying at sea for 2–4 days (Freduah et al., 2018; Owusu and Andriesse, 2022). The activities of SSF continue to evolve due to the increasing size of canoes, technological advancement, distance travelled, and targeted species (Smith and Basurto, 2019).

**Fig. 1:**
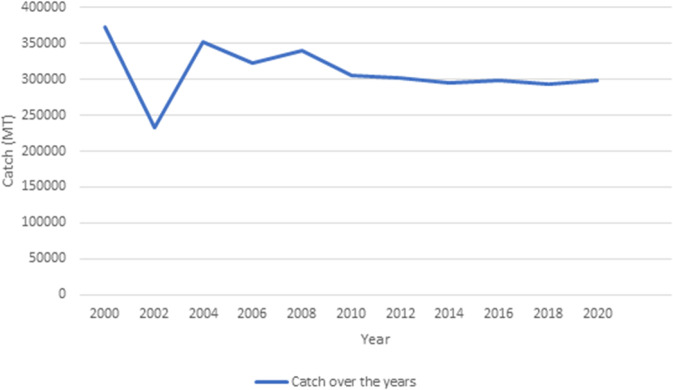
Marine fish production 2000–2020 in Ghana in metric tonnes. Source: Ministry of Fisheries and Aquaculture Development (MoFAD), Unpublished report (2019 and 2021).

The fisheries resources of Ghana have long been the backbone of local coastal communities’ economies, contributing significantly to socio-economic development (Crawford et al., 2016; Hasselberg et al., 2020; Adom et al., 2019; MoFAD, 2015). The SSF employs approximately 107,518 (primarily male) fishers and 1.9 million fish processors and traders (mainly female), accounting for about 80% of the total annual marine fish catch by volume (MoFAD, 2016). Fish is a preferred source of protein for most Ghanaians, making it critical for food security (MoFAD, 2016). In recent years, the capacity of the marine sector to support the livelihoods of coastal fishing communities has been under threat due to overfishing, illegal fishing activities, activities of industrial trawlers, climate change, and offshore petroleum production (Amadu et al., 2021; March and Failler, 2022; Adjei and Overå, 2019; Freduah et al., 2018; Owusu, 2019). The volume of marine fish production decreased from 373,000 metric tons in 2000 to 293,000 metric tons in 2018 (Fig. 1). The decline in fish catch has affected the number of fisherfolk working in artisanal fisheries. The number of fishers nationwide decreased from 139,155 in 2013 to 107,518 in 2016, representing a 22.7% decrease rate (MoFAD, 2016).

In response to the reduced marine fish stock, the Ministry of Fisheries and Aquaculture Development (MoFAD) implemented the closed fishing season policy in 2019 in consultation with diverse stakeholders, including the coastal fishing communities, NGOs (e.g. Friends of the Nation and Hen Mpoano), and civil organizations (e.g. Ghana National Canoe Fisherman Council, National Fish Processors and Traders Association). MoFAD is responsible for managing fish and other related fisheries products in Ghana. The Fisheries Act 2002 (ACT 625) and the Fisheries Management Plan (2014–2019) are two significant elements of the fishery management framework with specific policies that promote the sustainable harvest of marine resources. Since 2019, three closed fishing seasons have been enforced for the SSF sector. The first closed season was implemented between May 15 and June 15, 2019. The other two closed seasons were implemented in July 2021 and 2022. The closed season was not implemented by the authorities in 2020 due to COVID -19 pandemic (Graphic Online, 2022). The closed fishing season covers the entire 228,000 km^2^ Exclusive Economic Zone (EEZ) of Ghana’s coast.

Seasonal closures are an effective conservation tool in fisheries management to allow fish to reproduce during the spawning season before harvesting (Bucaram et al., 2018; Lazar et al., 2016; Bennett and Dearden, 2014; Rola et al., 2018). It aims to reduce the fishing intensity and protect target stock from mortality at a specific life stage (Samy-Kamal, 2015). In several countries, including Senegal, Philippines, Guinea Conakry, Mauritania, and Morocco, the closed season has successfully restored depleted marine fish stocks and improved marine sustainability (Lazar et al., 2016). However, even though closed fishing seasons can promote conservation and offer spill-over benefits, it is sometimes met with mixed perceptions and opposition, especially where it involves significant loss of livelihoods, as seen in Ghana and the Philippines (Owusu and Andriesse, 2020; Macusi et al., 2022).

To the best of the authors’ knowledge, there are no known studies concerning the factors influencing the compliance behaviour of the closed-season policy for the SSF in Ghana since its implementation in 2019. Ceptic and Nunan (2017) noted that non-compliance with fisheries management policies, such as temporal bans, may result in unsustainable fishing and decreased fish stock, negatively impacting fisheries’ livelihoods. This paper, therefore, investigates the factors that influence fisherfolk non-compliance with the temporal fishing bans in Ghana and suggests measures to improve compliance. The study contributes to the literature on marine conservation, fisheries management, coastal governance, and development studies by interrogating the viewpoint of fishers concerning the temporal ban on fishing. The empirical data gathered comprised 200 household surveys and 14 in-depth interviews with stakeholders at the local district, regional, and national levels. There is an anticipation that the results presented here will aid fisheries managers and stakeholders in making an informed decision regarding fisherfolk engagement in implementing future closed fishing seasons.

The rest of the paper is organized as follows: the next section presents the study’s theoretical framework, followed by the study areas and research methodology description. After that, the results and discussion are presented, highlighting the implications of our findings for improving coastal fisheries management, and finally, ends with the conclusion.

## Theoretical background

The literature on fisheries management concerning compliance comprises the instrumental and normative approach (Neilson, 2003; Ceptic and Nunan, 2017; Guirkinger et al., 2021). We adopt the definition of compliance presented by Agyekum (2016) as the act of obeying an order, a rule, or a request. The instrumental approach explains the cost–benefit analysis based on the assumption that individuals respond to the immediate benefits they could derive from compliance and non-compliance to any given situation (Neilson, 2003). Fishers are rational agents who make decisions by comparing the expected economic gains of non-compliance (Neilson, 2003; Ceptic and Nunan, 2017; Kraak and Hart, 2019). Therefore, the decision to obey or disobey a particular fisheries regulation influences the expected economic gains compared with the effectiveness of law enforcement, as well as the level of sanctions applied (Guirkinger et al., 2021; Al-Subni et al., 2013; Agyekum, 2016; Kraak and Hart, 2019). Studies suggest that artisanal fishers in Ghana violate effort-limiting regulations because of the low risk of detection and the low severity of the punishment (Akpalu, 2011; Agyekum, 2016). Fisherfolk’s non-compliance with fisheries regulations in the Sultanate of Oman was influenced by the lack of surveillance and poor enforcement of the rules (Al-Subhi et al., 2013), while low levels of sanctions contributed as an additional factor in Sudan (Abusin, 2012).

These findings demonstrate that fisherfolk’s non-compliance with fisheries management regulations is the balance between the potential monetary gains, the risk of being caught, and the severity of the sanctions. Therefore, the effectiveness of enforcing fisheries laws and the type of sanctions applied could influence compliance with fisheries management policies.

Fisher’s morality and perception of what is wrong and right can shape their behaviour and attitude concerning fisheries management regulations (Kraak and Hart, 2019; Nielson, 2003). The normative approach is a shared commitment towards marine conservation, unlike the instrumental or deterrence process, which makes compliance legal. The normative approach depends on personal convictions, social relations, informal social sanctions, perceptions of fairness, and the legitimacy of institutions (Guirkinger et al., 2021; Al-Subhi et al., 2013; Nielson, 2003).

Legitimacy is closely associated with the normative approach. Legitimacy in fisheries is the ‘acceptance by fishers of the applied regulations’ (Neilson, 2003, p. 427). Fishers will comply with the regulation statute if the law enforcement authorities and the law are seen as legitimate (Agyekum, 2016). Legitimacy in fisheries management influences the level of participation of fishers in the decision-making processes and the Fisher’s personal experiences with enforcement officers (Neilson, 2003; Guirkinger et al., 2021; Kraak and Hart, 2019). The acceptance and level of compliance with laws increase when local resource users, such as fisherfolk, are included in the decision-making processes relevant to marine conservation (Mikalsen and Jentoft, 2001; Msomphora et al., 2022). Al-Subhi et al. (2013) found that fairness in the application of rules and regular communication between fishers and fisheries managers are key factors that motivate fishers to comply with fisheries regulations in the Sultanate of Oman. However, in Peru’s giant oceanic manta ray fishery, compliance with fisheries regulations was hindered by the lack of perceived legitimacy towards authorities because of corruption and low social influence to comply (Guirkinger et al., 2021).

In this study, we applied selected factors derived from the instrumental and normative approach based on previous studies (Guirkinger et al., 2021; Neilson, 2003; Al-Subhi et al., .^2013^; Agyekum, 2016; Akpalu, 2011) to provide a holistic overview of the factors that influence compliance or non-compliance of the temporal bans on fishing in Ghana.

## Study area

**Fig. 2:**
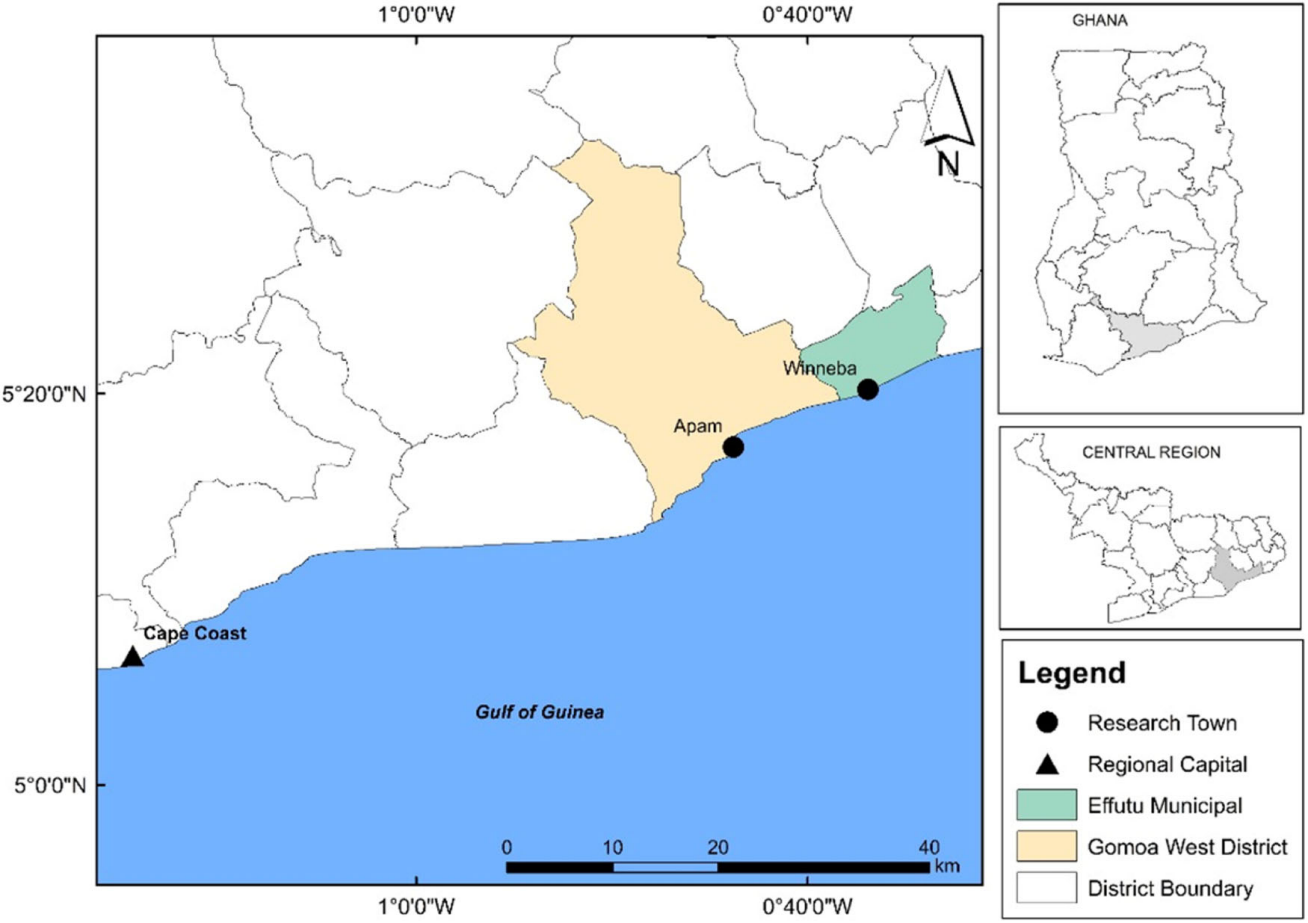
Map of the study areas in a regional and national context. Map showing the study areas in central region of Ghana.

Fishing is the main occupation of the people in the coastal areas of the Central Region, with 3855 canoes, and 97 landing beaches (MoFAD, 2016). The study focuses on the coastal towns of Winneba and Apam in the Central Region of Ghana (Fig. 2). These communities were selected because they have the country’s most active and largest fish landing sites, high dependence on fishing livelihoods, and recent concerns about declining fisheries.

Gomoa West District capital is Apam, approximately located 45 km east of the regional capital, Cape Coast, and lies between 5°18'17.91“N, 0°42'34.89“W, and 5°14'47.33“N, 0°46'35.27“W. The primary occupation of the people in the area is fishing (Ghana Statistical Services GSS, 2018). According to MoFAD (2016), 10 landing sites, 4062 fishermen, and 298 canoes are registered in the community to support the fishing industry. Mean fish production in the district over the past 5 years was 6308 tonnes per year (MoFAD, 2016). The primary fishing season in Apam waters is between August and December, and some of the commonly caught fish include threadfin (*Galeoides decadactylus*) and burrito (*Brachydeuterus auritus)* (MoFAD, 2016).

Winneba is a historic fishing port and the capital of Effutu District, lying 140 km east of Cape Coast on the south coast. Winneba’s fishing area lies between 5°19'37.95“N, 0°38'37.35“W, and 5°21'36.41“N, 0°34'29.07“W. The main occupations of the residents are agriculture and fishing (GSS, 2018). Winneba is a vibrant fishing area with five landing sites, 4270 fishermen, and 436 canoes (MoFAD, 2016). The small pelagics (*Sarinellas*) dominate the area, but occasionally the billfishes are also landed by the drift gill operators (MoFAD, 2016).

## Research methodology

### Data collection

The study used a mixed-methods approach to investigate fisherfolks’ compliance with the closed-season policy. The empirical data consist of a questionnaire survey of 200 SSF-dependent households and 17 interviews with stakeholders in the fisheries sector. The surveys and interviews were conducted between September and October 2021. The surveys were conducted in the communities of Apam in the Gomoa West District and Winneba in the Effutu District. The individual in-depth interviews (*n* = 17) were conducted with community leaders (*n* = 12), government officials (*n* = 4), and NGO officials (*n* = 1) to gain insights into the level of compliance.

The household questionnaire covered two broad thematic areas: socio-economic and demographic information and factors influencing compliance with marine conservation and fisheries regulations. The socio-economic and demographic section asked about the respondents’ level of education, age, fishing experience, income, fish landings changes, and the purpose of catch. From a management perspective, it is essential to have a clearer picture of the fishers’ demographics, knowledge, and professional experience (Al-Subhi et al., 2013). We derived questions from previous studies on compliance and non-compliance mechanism (See Table 2). The questionnaire included closed-ended, open-ended, and multiple-choice questions. The open-ended questions allowed the respondents to freely talk about their experiences and knowledge relevant to the local fishing industry and other relevant issues concerning the topic under investigation. The survey lasted between 50 min to an hour on average per person.

### Sampling method and sample size

Respondents for the household survey and interviews were selected by simple random and purposive sampling techniques, respectively. According to the 2016 national canoe frame survey, there were 4270 fishermen in Winneba and 4062 in Apam. The number of fishermen in the study communities was similar at the time of the survey. Information on the population of fishermen was sourced from the Chief fisherman to constitute the sample frame. A total of 200 fisherfolk were engaged in the research, including 120 randomly selected fishers engaged in small-scale commercial fishing from Winneba and 80 from Apam. The paper employed the purposive sampling technique to select community leaders, government officials, and an official from NGO. These groups of stakeholders were selected because of their knowledge and experience with coastal fishing communities. These sampling techniques are efficient data-gathering methods used by several authors to investigate fisheries livelihoods and coastal development in the Global South (Owusu and Adjei, 2021; Penney et al., 2017; Adjei and Overå, 2019; Marquette et al., 2002).

### Data analysis

Qualitative data obtained from interviews were transcribed, manually coded, and organized into relevant themes based on the study’s objectives. Selected narratives from the in-depth interviews were presented as direct quotations to illustrate key findings. The researchers used Microsoft Excel to organize the household data and SPSS Version 23.0 to undertake the statistical analysis. We employed descriptive statistics to summarize and present the results of the study. The Chi-square test for independence was used for the inferential statistical analysis to explore relationships between fisherfolk compliance with the closed season policy and the factors influencing their behaviour. We collected secondary data from published and unpublished reports, online news media, articles, and other internet sources to support the analysis of the study.

## Results and discussions

The survey report shows over 40% and 65% of respondents engaged in fishing over the past 20 years, and 93% and 88% reported a decrease in fish landing in Apam and Winneba, respectively (Table 1). The study found that 45.5% of respondents earn up to GHS500 per month, 37.5% earn between GHC 500–1000, and only 17% earn over GHS1000 per month.

**Table 1:** Key survey results (%).

Variable	Value	Apam	Winneba
Type of Fisher	Captain	13	22
	Crew member	79	62
	Net/canoe owner	8	16
Marital status	Single	40	26
	Married	54	67
	Divorced	6	7
Age	21–30 years	25	37
	31–40 years	33	36
	41–50 years	22	10
	51–60 years	20	7
	Above 60 years	--	10
Level of education	No formal education	86	71
	Primary education	14	29
Years of fishing experience	1–5 years	4	3
	6–10 years	15	10
	11–15 years	18	--
	16–20 years	20	21
	Over 20 years	43	66
Average monthly income	Up to 500 Cedis	44	47
	501–1000	37	38
	Over 1000 Cedis	19	15
Household size	One person	22	22
	2–5 persons	54	38
	6–10 persons	15	39
	More than ten persons	9	1
Catch quantity changes (over the past ten years)	Increase in catch	–	–
	Decrease in catch	93	88
	No change in the catch	7	12
Other jobs besides fishing	Yes	5	3
	No	95	98

Exchange rate: USD 1 = GHS 5.9 as of October 2021.

### Factors influencing compliance of closed fishing season

The results from the household survey, coupled with the interviews, confirm that instrumental and normative factors influence compliance with fisheries regulations.

Five main factors were identified regarding compliance with the temporal ban on fishing. These include; awareness and knowledge of the closed season, participation in the decision-making process before the implementation of the ban on fishing, involvement in the decision-making process during the ban on fishing, awareness of sanctions for breaching the ban on fishing, and acceptance of the closed season by traditional authorities. The results from Table 2 show that fisherfolk have good knowledge of the closed-season policy. The study revealed that fisherfolk’s awareness and understanding regarding the closed season is due to the involvement and participation of the fisherfolk in the decision-making processes before the introduction of the closed season (*M* = 4.60, SD = 0.70) and sustained engagement after the enforcement of the closed season. Fisherfolk mentioned that they interacted with the local fisheries leaders and municipalities. According to survey respondents, the regional fisheries leaders and municipal fishery officers cautioned them to desist from fishing during the ban and abstain from using unapproved gear for fishing. They reported that this interaction occurred during the distribution of compensation packages.

**Table 2:** Factors of compliance of closed season.

Variable	Mean	Std. D
Fishermen in this area know about the closed season	4.47	0.95
Participation in the fisheries management decision-making process before the implementation of the fishing ban	4.60	0.70
Participation in the fisheries management decision-making process during the fishing ban	3.05	1.86
Traditional authority acceptance of the closed season	4.18	0.91
Importance of fisheries compliance	1.97	1.37
Compensation for not fishing	1.57	1.25
The closed season would restore depleted fish stock	1.87	1.24
There are sanctions for fishers who fish during the closed season	4.10	0.84
Sanctions for breaching the closed season are enforced	1.71	1.35

Compliance factors (respondents’ agreement to the element are measured using a 5-point Likert scale, where 1.0 = strongly disagree, 2.0 = disagree, 3.0 = undecided, 4.0 = agree, 5.0 = strongly agree.

The first statement below illustrates the representation of fisherfolk in the decision-making process, and the second statement shows that community leaders expect fishers’ to comply with government policies:

A council, including the chief Fisher, chief fish trader, and selected canoe owners, represents all fishermen at various stakeholder engagements, where they convey the views of the local people to authorities (Fisher, Apam).

No fisherman from here ever dared to go to sea during the ban on fishing. The government placed the ban, and whether you like it or not, we have to obey, so everyone here complied with the closed season during the implementation (Fisher and community leader, Winneba).

These findings are consistent with results from previous studies (Al-Subhi et al., 2013; Guirkinger et al., 2021; Bose and Crees-Morris, 2009), which have established that the inclusiveness, active participation, and knowledge of the local resource users positively influence the compliance of temporal ban on fishing. Coastal management associated with top-down approaches, complex regulations, and non-transparent decision-making processes may decrease the credibility and legitimacy of fisheries regulations.

Results indicate that fishers know the consequences/sanctions for breaching the closed season (*M* = 4.1, SD = 0.84). The presence of laws is a deterrent to people who think of committing a crime. Sanctions are a deterrent only when perceived as more costly than the potential gains of breaking the law. Guirkinger et al. (2021) found that 76% of the surveyed fisherfolk were aware of the law banning manta ray fisheries in Peru; however, only 26% knew about the sanctions for non-compliance. Results from the study revealed no significant association between compliance with the closed fishing season and awareness of sanctions (*x*^2^ = 6.032, *p* = 0.644, Table 3). An indication that the understanding of sanctions is insufficient to cause fisherfolk to comply with the ban on fishing.

**Table 3:** Results of the chi-squared test (Fishers who comply and do not comply with the fishing ban).

Factors of closed-season compliance	Statistics
Fisherfolk awareness of the closed season	*x*^2^ = 22.362
	df = 8
	*p* = 0.01
Participation in the decision-making process before the ban on fishing	*x*^2^ = 19.232
	df = 4
	*p* = 0.000
Participation in the decision-making process during the ban on fishing	*x*^2^ = 58.993
	df = 6
	*p* = 0.000
Importance of fisheries compliance.	*x*^2^ = 5.872
	df = 8
	*p* = 0.662
The closed season would restore depleted fish stock (ecological effectiveness)	*x*^2^ = 11.312
	df = 8
	*p* = 0.185
Awareness of closed-season violation sanctions	*x*^2^ = 6.032
	df = 8
	*p* = 0.644
Enforcement of closed-season violation sanctions	*x*^2^ = 19.565
	df = 8
	*p* = 0.012
Traditional ‘authorities’ acceptance of closed season	*x*^2^ = 14.471
	df = 2
	*p* = 0.01
Compensation for not fishing	*x*^2^ = 18.565
	df = 8
	*p* = 0.013

*χ*^2^ = Chi square value obtained, df = degree of freedom, *p* = statistical significance at a confidence level of 95% (*p* < 0.05).

The acceptance of the closed season by community leaders (i.e. chief, elders, and other elected representatives) positively influences the compliance of fisherfolk (*M* = 4.18, SD = 0.91, Table 2). In coastal communities where fishing and its related activities remain the mainstay of the local economy, the chief fisherman commands good authority (Finegold et al., 2010). Before the introduction of the closed season by the government, other types of regulations were implemented by community leaders, as illustrated by the following statement:

Before the Ministry of Fisheries introduced the closed season, the traditional authorities were regulating overfishing with no fishing every Tuesday. Our community leaders convinced us that the closed season is a good way of marine conservation, just like the traditional bans on fishing (Fisher, Winneba).

The above finding demonstrates the relevance of local community leaders in marine conservation initiatives. Broader engagement with policies such as closed fishing season is more effective where there is greater buy-in to sustainable fisheries practices from the local community leaders. The findings from the study suggest that traditional leadership plays a critical role in ensuring compliance with fisheries regulations. Nunoo et al. (2015) argued that Chief Fishermen and Community Based Fisheries Management Committees are essential structures in the fisheries management system of Ghana. The assertion by Nunoo et al. (2015) was evident in the study areas as the respondents were vocal about the role of traditional management practices in the implementation and compliance with the closed season. Traditional conservation practices, such as implementing non-fishing days, have complemented government efforts to reduce overfishing. Traditional authorities also collaborate with other stakeholders, including national fisheries agencies, to ensure the incorporation of local views into fisheries management regulations (Bennett et al., 2001, 2019; Torell et al., 2019; Msomphora et al., 2022). In other jurisdictions, records show that traditional authority’s role in fisheries management is diminishing (Al-Subhi et al., 2013). It could have profound implications on fishers’ compliance with conservation initiatives. Guirkinger et al. (2021) found that fisherfolk have low social motivations to comply with fishing bans because of the limited involvement of the traditional authorities in fisheries management. The active involvement and participation of traditional authorities in marine conservation initiatives could increase the trust between national fisheries stakeholders and local resource users (Adjei and Overå, 2019).

### Factors influencing non-compliance

The key drivers of non-compliance with the closed season emanate from a perceived lack of ecological effectiveness, the lack of enforcement of sanctions, and the lack of compensation for loss of income during the fishing ban. A positive relationship was found between fisherfolk compliance with the closed season and the perception that they are ecologically effective (*x*^2^ = 11.312, *p* = 0.19). However, fisherfolk disagree (*M* = 1.87, SD = 1.24) with the assertion that temporal bans on fishing would contribute towards rebuilding the declining fish stock. This could negatively affect the effectiveness of the regulation because knowledge of relevance influences the effectiveness of policies (Kim and Kim, 2017; Kim and Oh, 2015).

Survey respondents reported that the one-month ban is too short for the fisheries to replenish. A recent study by Adom et al. (2019) found that the period for the closed season is too short to realize any significant increase in fish stock. In addition, fisherfolk pointed out that the closed season would not increase the fish stock because of the high levels of illegal fishing practices. This finding corroborates with Macusi et al. (2022), who found that temporal bans on fishing would not yield positive ecological outcomes without the corresponding enforcement of the laws on illegal fishing activities. Fisherfolk engaged in various forms of illegal fishing activities during the ban on fishing because of the perceived low risk of detection and the absence of severe sanctions for those found culpable (Neilson, 2003; Ceptic and Nunan, 2017). Owusu and Andriesee (2020) argued that the lack of law enforcement on illegal fishing in Ghana counteracts any potential benefits of fishing bans. The authors called on the government and other concerned stakeholders to invest more resources to combat illegal fishing activities.

Survey respondents complained about the lack of compensation schemes during the fishing ban. Most fisherfolks said they did not receive compensation from the government during the prohibition of fishing. The availability of compensation schemes received one of the lowest mean scores (*M* = 1.57, SD = 1.25) of the factors listed stimulating compliance with the fishing ban. The chi-squared test shows a positive relationship between closed-season compliance and compensation for not fishing (*x*^2^ = 18.565, *p* = 0.01). The significant association between closed-season compliance and payment for not fishing suggests that the closed-season policy would be successful when compensation packages and schemes are in place to support fisherfolks. Table 1 shows that most fisherfolks do not have any other jobs or livelihoods outside of fishing. More so, the results, as displayed in Table 1, show a significant reduction in catch quantity over the past decade with negative ramifications on the financial capital of fisherfolk. This situation makes it difficult for fishers to provide for their families. In Bangladesh, fishers who received compensation during the temporal ban on fishing abstained from illegal fishing (Bladon et al., 2018). However, Corrêa et al. (2014) found that due to the weak enforcement of the ban on fishing, fishers in the Brazilian Amazon continue to fish during the closed season despite receiving cash payments as compensation for abstaining from fishing. The reliance on fisheries as the primary source of livelihood to support families negatively affects compliance with fisheries regulations and marine conservation initiatives (Guirkinger et al., 2021).

Respondents disagreed with the statement indicating that sanctions for breaching the closed season are enforced (*M* = 1.71, SD = 1.35). Various studies have found that Ghana’s fisheries regulations are not efficiently enforced (Afoakwah et al., 2018; Agyakum, 2016). The lack of enforcement of the sanctions could compel fishers to engage in illegal fishing activities even during the ban on fishing (Guirkinger et al., 2021; Almeida Corrêa et al., 2014). The chi-squared test shows a positive relationship between closed-season compliance and enforcement of the sanctions for breaching the laws (*x*^2^ = 19.565, *p* = 0.01). The enforcement of fisheries regulations and fairness in applying the law are principal factors influencing fishers’ compliance with conservation initiatives (Al-Subhi et al., 2013).

### The implication of the study results toward improvement in fisheries management

Even though the findings from this study cannot be assumed to represent the situation in other coastal communities in Africa, the results provide two vital implications for marine conservation and coastal livelihood development. These are the co-management of conservation initiatives and the introduction of conservation payment schemes.

A co-management -made up of fisheries agencies and traditional authority committee members is a viable option to govern resources such as the ocean. The conventional conservation values and norms held in the community could result in high social acceptance of marine conservation-leading to the sustainable harvest of marine resources. It is worth mentioning that national fisheries agencies must demonstrate the political will to enforce sanctions on illegal fishing activities. Conservation measures are ineffective if they are not aligned with local development needs and complemented by a continuous drive toward reducing illegal fishing (Macusi et al., 2022).

The study suggests the introduction of conservation payment schemes alongside strict monitoring of the temporal ban on fishing. The conservation payment schemes could be in the form of cash payments or food items such as rice, sugar, cooking oil, etc., to cushion fishing households during the temporal ban on fishing. The Fishery officers must target the most vulnerable fishing households to achieve the objective of fisheries management (Bladon et al., 2018).

## Conclusion

This paper investigated the factors influencing fisherfolk behaviour concerning compliance with fisheries regulations. The results reveal that a combination of instrumental and normative factors affects fisherfolk’s compliance behaviour concerning the closed fishing season. Participation of fisherfolk and coastal communities in the fisheries management decision-making processes positively influenced compliance.

The key drivers of non-compliance with the prohibition on fishing emanate from a perceived lack of ecological effectiveness, the lack of enforcement of sanctions, and the lack of compensation during the ban on fishing.

This study focused mainly on two fishing communities in the Central Region of Ghana. Thus, future studies can consider multiple case study sites of coastal areas across the country using qualitative and quantitative approaches. Future studies should consider a comparative analysis of compliance behaviours between the SSF and industrial trawlers.

## Data Availability

The datasets generated during the study are available upon request from the corresponding author.
